# Molecular dynamics simulations of the sputtering of boron and boron oxide surfaces†

**DOI:** 10.1039/d5ra05589j

**Published:** 2025-09-19

**Authors:** Shokirbek Shermukhamedov, Thana Maihom, Kersti Hermansson, Michael Probst

**Affiliations:** a Department of Chemistry, Ångström Laboratory, Uppsala University 75121 Uppsala Sweden shokirbek.shermukhamedov@kemi.uu.se; b Institute of Ion Physics and Applied Physics, University of Innsbruck Technikerstraße 25 6020 Innsbruck Austria michael.probst@uibk.ac.at; c Division of Chemistry, Department of Physical and Material Sciences, Faculty of Liberal Arts and Science, Kasetsart University Kamphaeng Saen Campus Nakhon Pathom 73140 Thailand

## Abstract

We employ classical molecular dynamics (MD) simulations to study processes governed by particle–surface interactions. The interatomic potential energy functions are described by a neural network potential (NNP) trained on an extensive set of density functional theory (DFT) calculations in a semi-iterative fashion. Potential construction and simulation set up follow the Behler–Parrinello approach. As a specific example we investigate sputtering, reflection, and adsorption phenomena occurring on boron and boron oxide surfaces under the impact of deuterium atoms, systems that reflect recent developments in materials science. Besides the frequent use of boron surfaces as an oxygen-gathering material in technical applications, boron-based compounds will be used in future fusion devices. Understanding their interaction with energetic plasma particles is essential, yet their stability and sputtering behavior at the atomic level have remained largely unexplored. From our simulations, we analyzed sputtering yields, adsorption, and reflection events on both boron and boron oxide surfaces. The production simulations included about 750 atoms and covered a range of incident energies and impact angles. Increasing the deuterium impact energy generally leads to an increase in sputtering yields but with distinct energy-dependent trends. The sputtering yield of boron from boron oxide surfaces remains significantly lower than from surfaces of pristine boron. We use an analytical approach to estimate an effective surface binding energy. The work presented here, especially the various energy- and angle dependent rates, can be used to create a parametric model of the B and B_2_O_3_ surfaces under the impact of hot particles.

## Abstract 2

Sputtering yields of atoms from boron and boron oxide surfaces as a function of the impact energy of deuterium.

## Graphical abstract

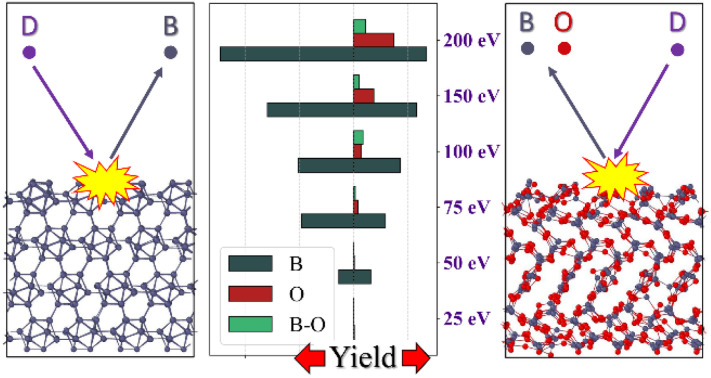

## 1 Introduction

The interactions between surfaces and their environment are crucial for understanding the degradation of materials and devices in various applications, ranging from semiconductor manufacturing to nuclear fusion.^1–3^ In thermonuclear devices, particularly in ITER, the interaction between the plasma particles and plasma-facing materials (PFM) is important in determining the efficiency, lifetime, and safety of the fusion process.^4^ Among the components most exposed to the plasma environment are the divertor and the first wall of the blanket. These materials must withstand extreme heat loads, high particle fluxes, and continuous irradiation by energetic plasma particles such as deuterium (D), tritium (T), helium (He), and many other ions present in the plasma.^5^ During the fusion operations, PFMs can degrade the structural integrity and lead to the contamination of the plasma with impurities. To reduce these effects, materials with low atomic numbers (low-*Z*), like boron and beryllium, are important due to their low sputtering yields, low hydrogen retention at higher temperatures, and their smaller loss of energy when they enter the plasma.

Boron and its compounds, particularly boron trioxide (B_2_O_3_), have attracted attention as potential candidates for use in PFM protective layers. Boronization – depositing boron onto PFM surfaces – is routinely applied in several experiments to reduce impurity influx and improve plasma confinement, taking advantage of both the low nuclear weight and the oxygen affinity of boron.^6–10^ Sputtering of boron hydrates under the impact of hydrogen isotopes is called chemical sputtering. Such molecule formation processes may cause larger degradation of boron-containing surfaces than purely physical sputtering. The detailed atomistic mechanisms governing the sputtering and surface evolution of boron-based materials under deuterium bombardment are not yet fully understood.

A large number of theoretical and computational studies have been devoted to the sputtering of materials under various conditions.^11^ Fitting experimental sputtering yields using empirical or semi-empirical formulas, such as the Eckstein^12^ or Yamamura^13^ models, allow for the interpretation and extrapolation of experimental data. Quantum chemical calculations, particularly DFT and the various AIMD schemes, are the most accurate methods among computational techniques, allowing analysis of sputtering events, surface binding energies, energy transfer, and mechanisms of sputtering during particle impacts, although they remain computationally expensive and impractical for large-scale and/or long-time simulations.

Classical MD simulations with simple analytical potentials are fast and can model large systems.^14^ However, their accuracy depends strongly on the quality of the interatomic potentials. Recent progress in machine learning, especially neural network potentials (NNPs) trained on DFT datasets, now allow simulations that retain DFT-level accuracy with near-classical MD efficiency.^15,16^ These NNP-based MD simulations are becoming essential in sputtering research, bridging the gap between computational cost and predictive accuracy for complex surface and plasma–wall interaction phenomena. Simulations with the Monte-Carlo based Binary Collision Approximation (BCA) method offer statistical predictions over larger ensembles and longer timescales but often require fitting parameters and miss atomistic detail.^17^

Many studies in the sputtering field have focused on fitting interatomic potentials and on investigating the erosion behavior of boron and its compounds. Domínguez-Gutiérrez and Krstić^18^ investigated the erosion of boron and boron carbide in contact with deuterium plasma using reactive force fields. They calculated sputtering yields under low-energy particles (up to 30 eV) and found a significant effect of chemical sputtering in boron and boron carbides. Deng and Du^19^ developed and validated composition-dependent empirical potentials specifically for boron oxide in multicomponent glasses, enabling large-scale MD studies of B_2_O_3_ structural dynamics and rare atomistic configurations. However, accurate interatomic potentials for B/B_2_O_3_-plasma particle are still lacking.

In this paper MD simulations of sputtering processes from boron and boron oxide under deuterium bombardment are performed at various incident energies and impact angles. A specifically created neural network potential drives the simulations. The data and findings presented below contribute to an understanding of surface erosion processes, enhancing the development of materials for future fusion applications. The simulation trajectories provide detailed information of incident particle reflection, retention, and adsorption ratios as functions of impact energy and angle, together with effective surface binding energies (*E*_SB_), allowing insight into the general field of surface interactions under particle bombardment.

## 2 Methods

### 2.1 Behler–Parrinello method

In the Behler–Parrinello approach the total energy of the system is expressed as the sum of its atomic energies *E*_i_.^20^ The atomic energies are derived from element-specific, atom-centered high-dimensional neural networks (HDNN). The Cartesian coordinates of the neighbors of each atom are transformed into weighted radial and angular symmetry functions, to ensure invariance against rotation, translation and exchange within the same atomic species. A cut-off radius of 7 Å restricts the environment of each atom. The atomic coordinates were transformed through symmetry functions, using parameters provided in Tables S1 and S2 of the SI, and served as input for the feedforward neural network. The network had two hidden layers with 25 nodes each, using soft-plus activation functions and offsets to predict the material energies. Once the parameters of the NNPs are trained (see Section 2.3), the MD simulations are conducted using a modified LAMMPS code.^21,22^

### 2.2 Generation of training data

Density functional theory calculations generate the energies and forces used to train and test the NN parameters. The Behler–Parinello formalism thus acts as a bridge to produce DFT-quality forces in the simulations which are much faster than it would be possible with direct DFT. Initial training and test sets are generated from systems where the atomic positions are allowed to slightly deviate from their DFT-optimized equilibrium structures. Here, after a first training, new data were generated from actual sputtering simulations. This yielded trajectories with various incident angles of deuterium on small supercells. During these simulations, the lowest atomic layer was fixed, while other atoms were allowed to move unrestricted. The collected structures from these runs were recomputed at the DFT level. In all DFT calculations we employed the PW91 functional^23^ within the generalized gradient approximation (GGA) framework as implemented in the Vienna *Ab Initio* Simulation Package (VASP).^24,25^ A large supercell and a Gamma-centered *k*-point mesh of 3 × 3 × 3 were used with a plane wave basis set and a cut-off energy of 350 eV. The convergence threshold of the forces was set to 10^−4^ eV Å^−1^. The simulations were spin-unpolarized. This methodology is consistent with previous studies on the interaction of plasma particles with various targets.

### 2.3 Network training

The training data were then used to train Neural Network Potentials for the boron–deuterium (B(100)–D)^26^ and boron-oxide–deuterium (B_2_O_3_(001)–D)^27^ systems. The final reference datasets include a mix of surface and bulk structures, with 4861 configurations for the pure boron systems and 5695 for the boron oxide systems, containing a total of 655 433 and 605 454 atoms, respectively. Of these, 90% of the structures were used to train the final NNP, while the remaining 10% were used for testing. After 100 training steps, the model achieved root-mean-square errors (RMSE) of 6.72 and 10.1 meV per atom for the predicted energies, and 0.43 and 0.66 eV Å^−1^ for the atomic forces in the boron and boron oxide test sets, respectively.

**Fig. 1:**
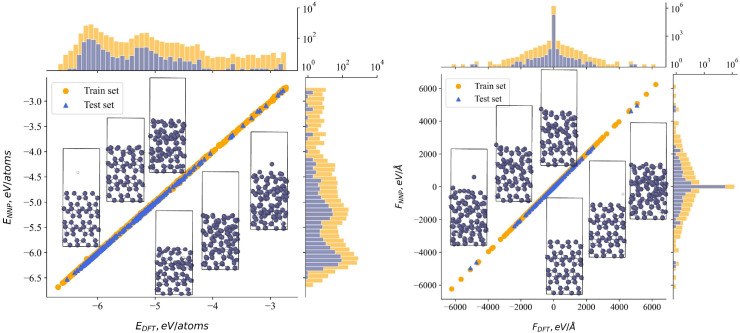
Correlation between DFT-calculated and NNP-predicted atomic energies (left) and forces (right) for the boron–deuterium dataset, accompanied by histograms illustrating the distribution of their values. The structures shown below and at the bottom scatter points provide representative examples corresponding to the distributions presented above.

Fig. 1 shows the accuracy of the NNP with respect to the DFT data together with the distribution of data points for the B–D dataset; the corresponding plots for the B_2_O_3_–D dataset, are presented in Fig. S1 (SI). Considering the ranges of both energies and forces, the figures confirm that indeed the DFT data is carried over to the NNP model with no visible loss of accuracy. The expression to create the symmetry-adapted input for the NNP are given in the SI.

Further validation of the trained NNPs was done by calculating the surface atom removal energy and the adsorption curves of deuterium atoms on boron (Fig. S2, SI) and boron oxide (Fig. S3, SI) surfaces. These calculations also served as cross-validation tests of the trained NNPs, allowing us to estimate the RMSE of models on these structures. The obtained RMSE values for energies and forces are given in Tables S3 and S4 (SI). The results show that the RMSE values are lower than those from the training dataset tests, which confirms that the NNPs can be reliably used for sputtering simulations.

### 2.4 MD simulation protocol

**Fig. 2:**
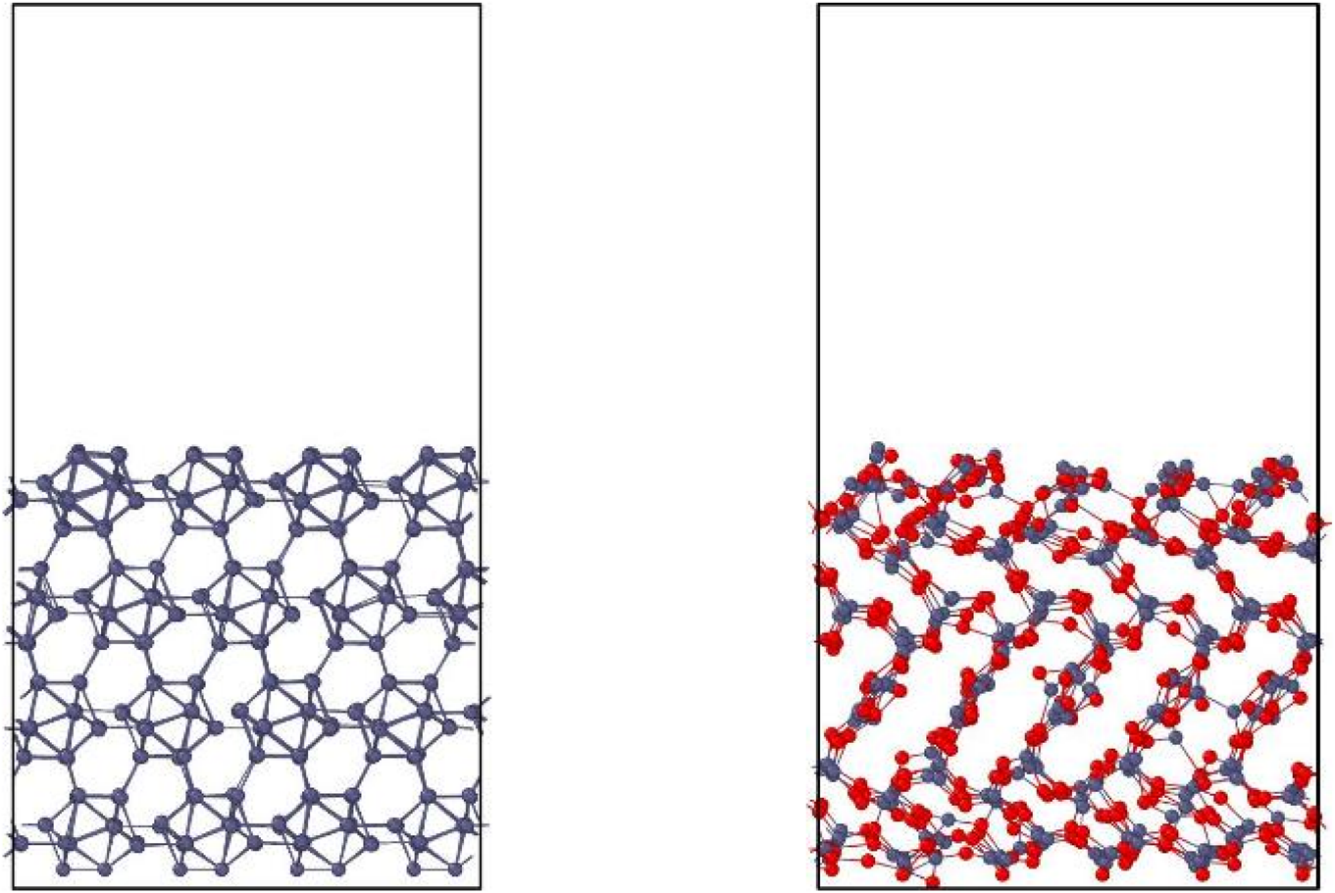
Simulation cells used in the non-cumulative MD simulations. Left: B(100); right: B_2_O_3_(001).

Molecular dynamics can simulate sputtering in two ways: cumulative (many impacts in one simulation) or non-cumulative (one impact in one simulation). The cumulative case resembles real processes, while the non-cumulative case gives clearer statistics by isolating single impacts without accumulated damage. Employing the trained NNPs, we performed non-cumulative MD simulations of bombardment by deuterium (D) projectiles with energies of ranging from 20 to 200 eV at impact angles of 0°, 30°, and 60°. 2000 simulations for each set of parameters were performed and the resulting trajectories were used in the statistical analysis of the events. The initial simulation boxes were created from B_768_ and (B_2_O_3_)_150_ surfaces, oriented along the *z* direction. The box dimensions (in *x*, *y*, *z*) were 19.60 × 17.05 × 32.1 Å for the boron system and 21.80 × 18.80 × 36.8 Å for the boron oxide system (Fig. 2). Initially, the atomic positions were relaxed at 300 K in the NVT ensemble for 0.1 ps. The obtained surfaces were then used for sputtering simulations in the NVE ensemble. The D atoms as projectiles were deposited as described in Section 2.2. The timestep was dynamically adjusted between 10^−4^ and 1 fs depending on the velocity of the projectile, with the step size being resampled every five steps such that no atom moved more than 0.05 Å between these five steps. The simulation proceeds until one of several halting conditions is met.

## 3 Results

### 3.1 Sputtering yields of boron

**Fig. 3:**
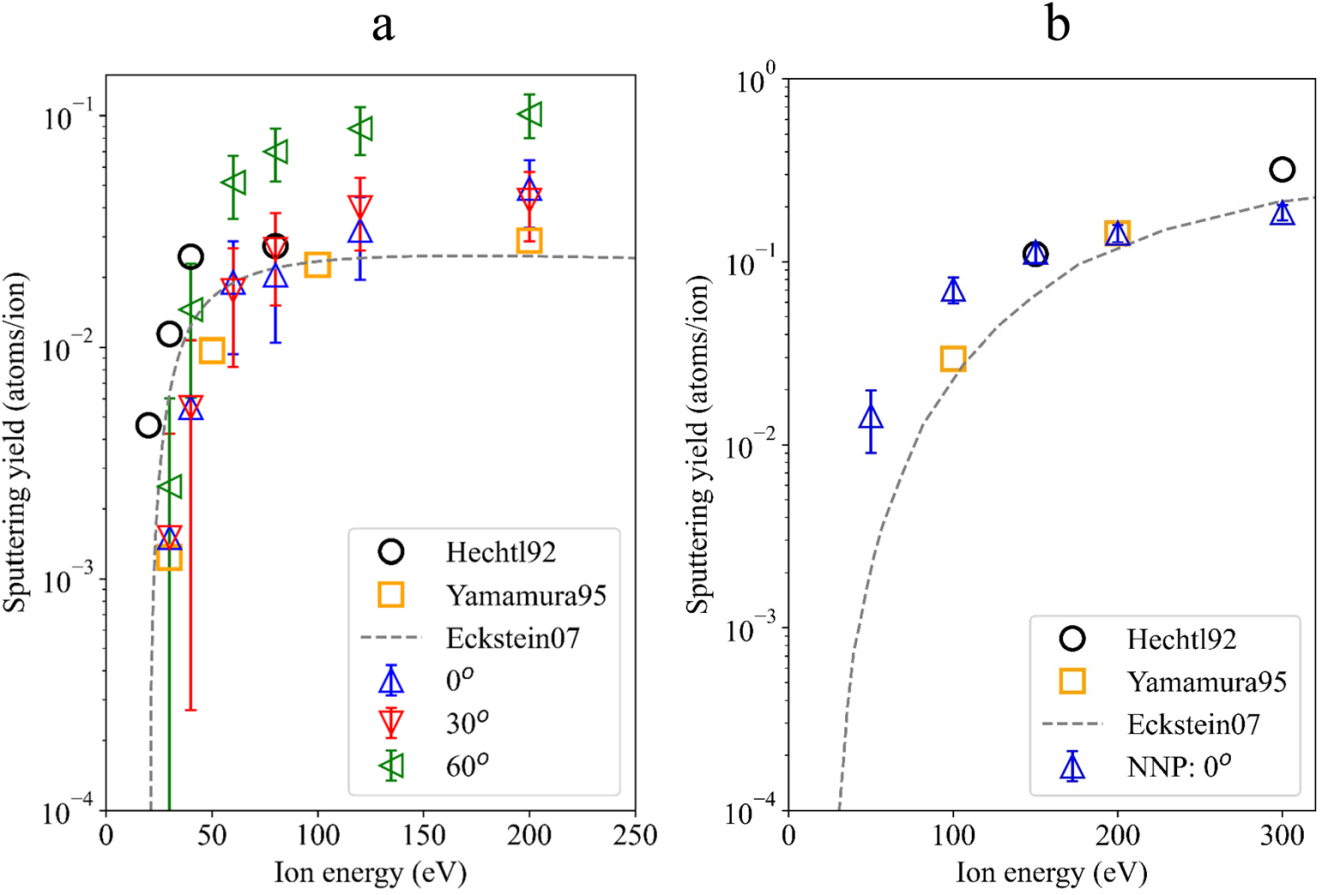
Sputtering yields of boron from a boron surface under bombardment by deuterium (a) and boron atoms (b). Triangles represent our NNP simulations. 0° means perpendicular impact.

In Fig. 3a the sputtering yields of boron from a pure boron surface under deuterium bombardment are shown together with the available literature data. The experimental data from Hechtl *et al.*^28^ and computational data from Yamamura,^13^ along with the Eckstein curve^29^ are included. Our values for different incident angles are indicated by colored triangles. It is seen that at perpendicular impact the yields increase as the impact energy increases. The statistical error of the sputtering yield was estimated using a bootstrapping procedure,^30^ where random resampling with replacement was applied to the set of MD trajectories. The variance of the resampled averages was then used to calculate the uncertainty of the sputtering yield.

At lower impact energies our sputtering yields are smaller than the experimental and computational benchmark points. The large error bars for our data points are caused by the underlying Poisson statistics due to infrequent events. Increasing impact angles causes higher sputtering yields. At 60° the highest sputtering yields are found. They reach a plateau above the benchmark points, while for 30° a small maximum at 120 eV occurs with a decline for higher energies.

In addition to deuterium bombardment, we investigated the self-sputtering of boron surfaces using also the B–D trained NNP. The results, presented in Fig. 3b, show the sputtering yields of boron when bombarded by boron atoms. The agreement between the present results (blue triangles) and the former experimental^28^ and computational^13^ data, as well as with Eckstein's curve is much better, probably due to better training data and the system involving only one element. These B self-sputtering simulations were only performed for perpendicular impact.

### 3.2 Sputtering of boron oxide

**Fig. 4:**
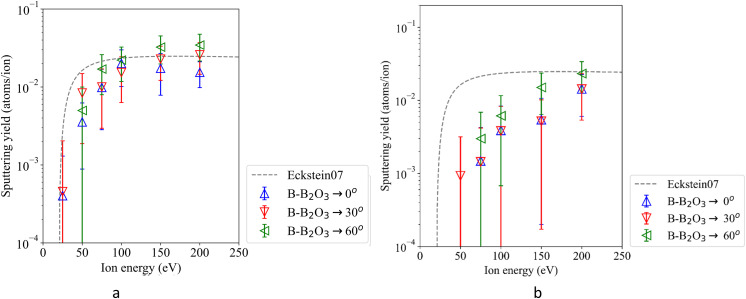
Sputtering yields of boron atoms (a) and oxygen atoms (b). For D colored triangles indicate different incident angles. The Eckstein curve given for reference shows yields for pure boron surfaces only.

As mentioned, boron surfaces often serve the purpose of gathering oxygen, with boron oxides being formed. Simulations of the sputtering of a B_2_O_3_ surface by deuterium provides insight into the mechanism of sputtering of boron and oxygen atoms from them. The sputtering yields of boron atoms are illustrated in Fig. 4a, with different triangles representing the incident angles. The Eckstein curve for pure boron surfaces is included for comparison as it is also in the oxygen sputtering panel (Fig. 3b). The highest yield is observed at an impact angle of 60°. At an angle of 30°, the boron sputtering yield reaches a maximum at 200 eV. Below 150 eV, the calculated sputtering yields of boron from B_2_O_3_ surface are seen to be lower than those from pure boron surfaces. Oxygen atoms form strong bonds with boron, creating a robust surface that requires higher energy to remove boron atoms.

We have also analyzed oxygen sputtering from B_2_O_3_. Fig. 4b shows the yields of oxygen atoms. The yields of oxygen atoms increase monotonously with the energies and angles. Initially, the oxygen sputtering yields are lower than those of boron, but they become comparable at 200 eV. The differences between boron and oxygen can be attributed to the energy transfer factor:^29^*γ* = 4*M*_1_*M*_2_/(*M*_1_ +*M*_2_)^2^ where *M*_1_ and *M*_2_ are the atomic masses of the projectile and target atom, respectively. Boron has a lower mass compared to the oxygen; accordingly, boron sputtering yields become higher when the surface contains both elements.

The results also show the significant effect of the impact angle on sputtering yields. At higher impact angles, the projectiles transfer some of their energy near the surface, increasing the probability of atom sputtering. This results in higher yields compared to normal incidence (0°). Cases with normal (=perpendicular) impact led to deeper penetration (details in Section 3.4) and less efficient sputtering. This is especially true at high energies, above 200 eV, where projectiles can penetrate deeper into the material and generate collision cascades that extend beyond the finite simulation cell. Consequently, part of the energy transfer and defect production is not fully captured, which can lead to underestimated sputtering yields and inaccurate defect statistics. Such effects can be more accurately described by cumulative simulations.^31^

### 3.3 Reflection, adsorption and retention probabilities of the projectile

**Fig. 5:**
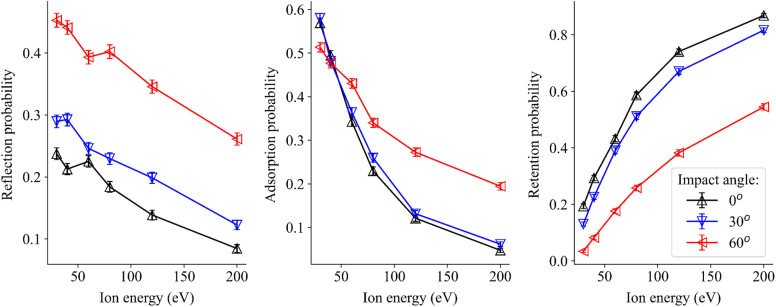
Deuterium atoms after collision with a boron surface: probabilities of reflection (left), adsorption (middle), and retention (right).

While sputtering yields are readily measurable, for example by weight loss of the surface and an obvious sign of degradation, other processes take also place if D atoms interact with the surfaces. We analyzed D reflection probabilities, probabilities of D adsorption on the surface and retention of deuterium in the bulk. This is especially important as it can lead to the formation of boranes and boron hydrides and, *via* oxygen–hydrogen interactions, to the formation of hydroxyl groups. These chemical modifications can increase surface degradation of PFMs. We find that lower projectile energies, reflection and adsorption phenomena are dominant (Fig. 5), as is evident from the relatively low sputtering yields.

At higher energies, the deuterium atoms possess sufficient kinetic energy to penetrate deeper into the samples, causing more collision cascades and can effectively distort the surface structure. Collision cascades can also create subsurface damage and vacancies, further contributing to the sputtering processes. This is true for different impact angles even though the probability of reflection and adsorption is higher at larger incident angles. At normal incidence (0°), the projectiles penetrate the surface more efficiently.

Differences between the two surfaces manifest themselves mainly in the reflection probabilities. Reflection of deuterium from boron oxide (Fig. S4 in SI) is lower than from pure boron surfaces. This is not unexpected, since with D implanted in B_2_O_3_, strong oxygen–deuterium interactions can appear. This in turn supplies energy for bond breaking and for chemical reactions, which can be observed indirectly by the increased sputtering yields in B_2_O_3_ compared to pure boron.

### 3.4 Energy profile of sputtered boron atoms

**Fig. 6:**
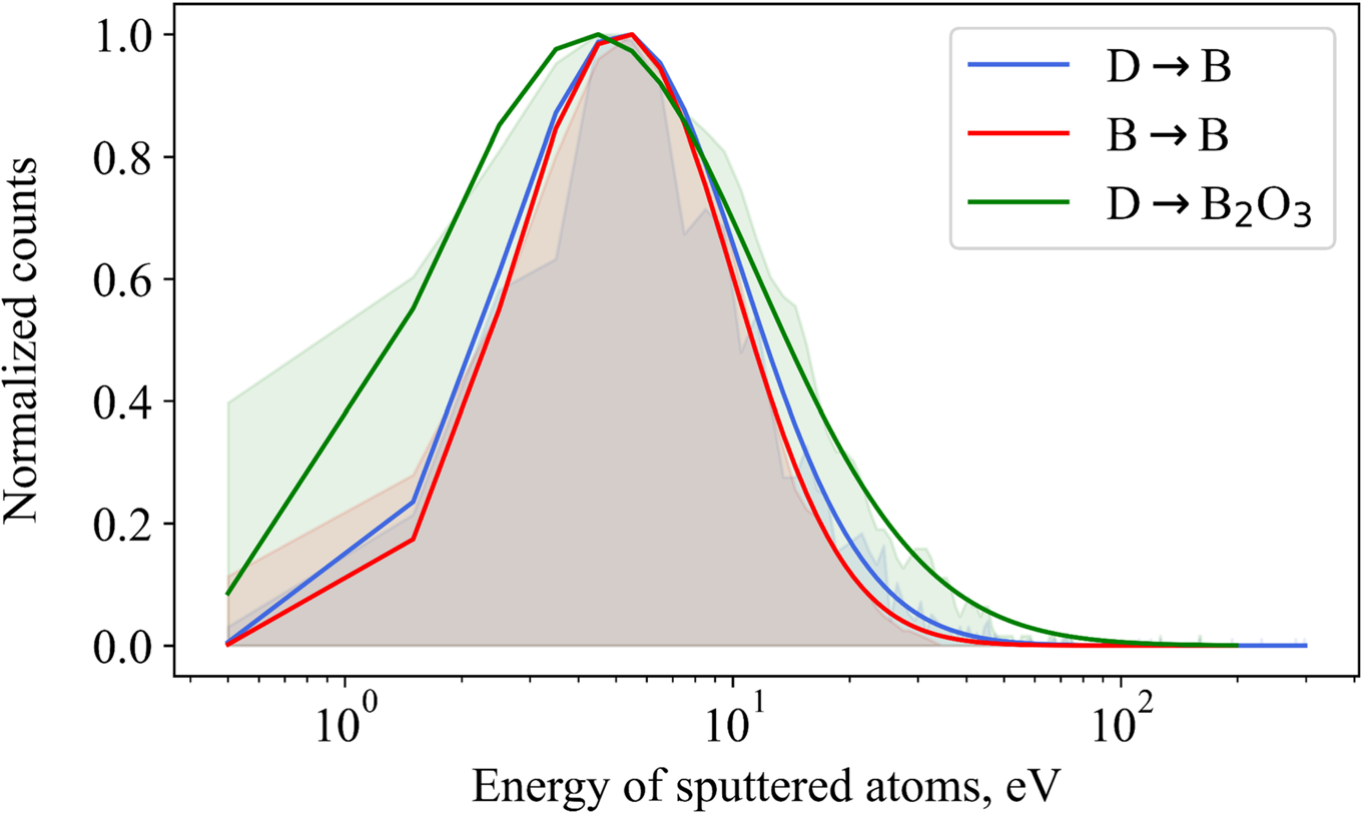
Energy distributions of sputtered boron atoms. The shaded areas represent the normalized yield. The solid lines are lognormal distributions fitted to the data.

The energy distribution of boron atoms which have been sputtered from a boron surface by either D or B particles, or by D from a B_2_O_3_ surface, is illustrated in Fig. 6 where all outgoing energies and angles are sampled together. The shaded areas under the distributions represent their normalized yields. It turns out that these energy profiles closely follow a lognormal distribution^32^ which is represented by the solid lines. The positions of the maxima in the final fit to such a distribution approximate the surface binding energy, defined as the amount of energy required to remove an atom from the surface. The effective surface binding energy (*E*_SB_) and indeed the energy distribution of sputtered atoms itself is important because it can be used to predict how far they spread out into the plasma and where they are possibly prone to surface re-deposition.

The effective surface binding energy for boron sputtered from deuterium impact was found to be 5.14 eV; the reference value of this quantity in a BCA simulation was, for example, taken as 5.73 eV.^29^ Under self-bombardment conditions, the effective *E*_SB_ for boron was 5.15 eV, closely matching the value from deuterium bombardments. This consistency suggests that the underlying atomic-scale bonding characteristics of the boron surface remain the dominant factor influencing the effective *E*_SB_, even for different projectile masses and energy transfer dynamics.

Similarly, sputtering of boron oxide by D can be used to extract effective surface binding energies of boron and oxygen, leading to *E*^B^_SB_ = 4.46 eV and *E*^O^_SB_ = 3.53 eV, respectively. This effective *E*_SB_ for boron is 10% less than a pure boron surface. This difference can be explained in terms of the energy transfer value as well. However, the joined B and O energy spectra (green line in Fig. 6) give a value of 4.36 eV, which means the contribution of oxygen to the total sputtering yield is negligible. Thus, from the viewpoint of sputtering, boron determines the effective surface binding energy of the oxide surface.

### 3.5 Chemical sputtering

**Fig. 7:**
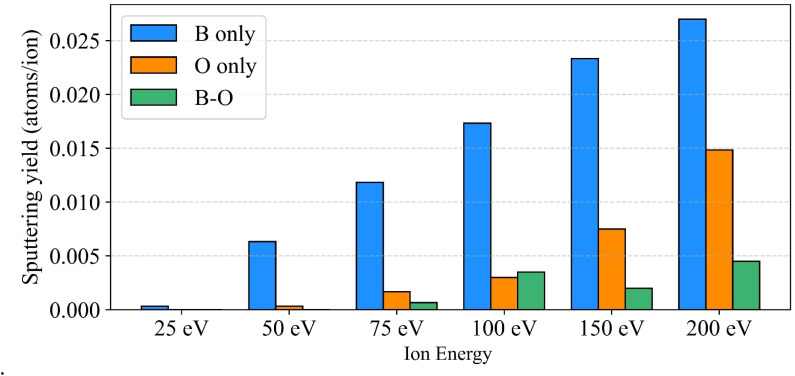
Sputtering yields of B, O and B–O from B_2_O_3_ surfaces under deuterium bombardment.

Another important process possibly influencing the effective *E*_SB_ values is chemical sputtering. Both boron and boron oxide surfaces can strongly interact with hydrogen isotopes, which can lead to the ejection of hydrides and alter both the sputtering mechanism and surface composition. To investigate this, we analyzed the sputtering of B–B, B–D, B–O, and O–D molecular fragments. We find that the sputtering yields of B–D and O–D molecules each contribute less than 5% to the total sputtering yield. On the other hand, B–O sputtering becomes significant at low impact energies, reaching up to 20% of the boron sputtering yield. Fig. 7 shows energy-resolved sputtering yields of B, O and B–O sputtering events. Oxygen-induced chemical sputtering reaches a maximum around 150 eV, highlighting its role in modifying the sputtering behavior at intermediate energies. The relative importance of chemical sputtering is visible from Fig. 7 as it increases with increasing energy up to 150 eV. One could expect that chemical sputtering would be even more prominent in the scenario of cumulative simulations where adsorbed deuterium molecules from former irradiation events are present.

## 4 Conclusions

Employing HDNNP-based MD simulations, we have investigated the sputtering of boron and boron oxide under deuterium bombardment, as well as boron self-sputtering on pure boron surfaces. Simulations with various impact angles and energies allowed us to analyze sputtering yields and related quantities. Sputtering from boron oxides shows element-specific behavior. Energy spectra of the sputtered atoms were computed, allowing estimation of the effective surface binding energies. The *E*_SB_ values for boron oxide were found to be lower than those of pure boron. However, the sputtering yields of boron from the oxide surface remain comparable to those from the pure boron surface. Additionally, on the oxide surface, chemical sputtering involving the formation of B–O radicals occurs. Along with the computed probability densities, these data are useful for predicting the behavior of boron–oxygen surface layers and assessing the long-term stability of boron-based plasma-facing materials.

## Author contributions

S. S.: investigation, formal analysis, data curation, writing, visualisation M. P.: conceptualization, funding acquisition, methodology, administration T. M.: resources, writing K. H.: validation, resources, writing.

## Conflicts of interest

There are no conflicts of interest.

## Supplementary Material

RA-015-D5RA05589J-s001

## Data Availability

The data supporting this article have been included as part of the SI. See DOI: https://doi.org/10.1039/d5ra05589j.
