# Supplementary material for: Molecular dynamics simulations of the sputtering of boron and boron oxide surfaces
Source: RSC Adv. 2025 Sep 19;15(41):34274–81. doi: 10.1039/d5ra05589j (PMC12447261; doi:10.1039/d5ra05589j)
Supplement: RA-015-D5RA05589J-s001 [file RA-015-D5RA05589J-s001.pdf]

Supplementary Information (SI) for RSC Advances.
This journal is © The Royal Society of Chemistry 2025

Supplementary Materials

Molecular dynamics simulations of the sputtering of boron and boron oxide surfaces

S. Shermukhamedov[1,2], Thana Maihom[3], Kersti Hermansson[1] and Michael Probst[2]

[1] Department of Chemistry, Ångström Laboratory, Uppsala University, 75121 Uppsala, Sweden

[2] Institute of Ion Physics and Applied Physics, University of Innsbruck, Technikerstraße 25, 6020 Innsbruck, Austria

[3] Division of Chemistry, Department of Physical and Material Sciences, Faculty of Liberal Arts and Science, Kasetsart University, Kamphaeng Saen Campus, Nakhon Pathom 73140, Thailand

1. Symmetry functions

Radial and -angular Behler-type weighted atom-centered symmetry functions are defined in Eqns. (1) and (2), respectively, and are the same as in Refs.[1,2].

$$G_i^{rad} = \sum_{j \neq i} g(Z_j) e^{-\eta(R_{ij} - R_s)^2} f_c(R_{ij}) \qquad (1)$$

$$G_i^{ang} = 2^{1-\zeta} \sum_{i \neq j}^{N} \sum_{k \neq i,j}^{N} h(Z_j, Z_k)(1 + \lambda \cos\theta_{ijk})^{\zeta} e^{-\eta(R_{ij} - R_s)^2} e^{-\eta(R_{ik} - R_s)^2} e^{-\eta(R_{jk} - R_s)^2} f_c(R\text{[illegible]} \qquad (2)$$

The parameters of radial and angular symmetry functions employed to describe the local atomic environments in the input layer of the neural network for three elements (B, O and D) are listed in Tables S1 and S2.

Table S1: Parameters of radial symmetry functions employed to describe the local atomic environments in the input layer of the two elements neural network (B, O, D). r_{cut} is the cutoff radius and the meaning of the rest parameters refer to the definitions in articles[1,2].

η	R_S	r_{cut}
**1.31E+00**	0.00E+00	1.30E+01
**1.31E+00**	6.19E-01	1.30E+01
**1.31E+00**	1.24E+00	1.30E+01
**1.31E+00**	1.86E+00	1.30E+01
**1.31E+00**	2.48E+00	1.30E+01
**1.31E+00**	3.10E+00	1.30E+01
**1.31E+00**	3.71E+00	1.30E+01
**1.31E+00**	4.33E+00	1.30E+01
**1.31E+00**	4.95E+00	1.30E+01
**1.31E+00**	5.57E+00	1.30E+01
**1.31E+00**	6.19E+00	1.30E+01
**1.31E+00**	6.81E+00	1.30E+01
**1.31E+00**	7.43E+00	1.30E+01
**1.31E+00**	8.05E+00	1.30E+01
**1.31E+00**	8.67E+00	1.30E+01
**1.31E+00**	9.29E+00	1.30E+01
**1.31E+00**	9.91E+00	1.30E+01
**1.31E+00**	1.05E+01	1.30E+01
**1.31E+00**	1.11E+01	1.30E+01
**1.31E+00**	1.18E+01	1.30E+01
**1.31E+00**	1.24E+01	1.30E+01
**1.31E+00**	1.30E+01	1.30E+01

Table S2: Parameters of angular symmetry functions employed to describe the local atomic environments in the input layer of the two elements neural network (B, O, D). r_{cut} is the cutoff radius.

η	R_S	λ	ζ	r_{cut}
**5.56E-02**	1.00E+00	-1	1	1.30E+01
**5.56E-02**	1.00E+00	1	1	1.30E+01
**5.56E-02**	4.00E+00	-1	1	1.30E+01
**5.56E-02**	4.00E+00	1	1	1.30E+01
**5.56E-02**	7.00E+00	-1	1	1.30E+01
**5.56E-02**	7.00E+00	1	1	1.30E+01
**5.56E-02**	1.00E+01	-1	1	1.30E+01
**5.56E-02**	1.00E+01	1	1	1.30E+01
**5.56E-02**	1.30E+01	-1	1	1.30E+01
**5.56E-02**	1.30E+01	1	1	1.30E+01

2. NNP validation

To validate the thus trained NNP, adsorption curves of D on boron and its oxide surfaces were calculated. Additionally, we determined the energy required to remove different surface atoms at various surface positions. Figures S2 and S3 show these graphs. Curves show that the NNP is able to predict structures not included in the training set, especially around the minima. However, at higher distances, the NNP struggles to match the DFT points and shows some shift, which can be attributed to the lack of such structures in the training dataset. Tables S3 and S4 shows RMSE for these validation calculations.

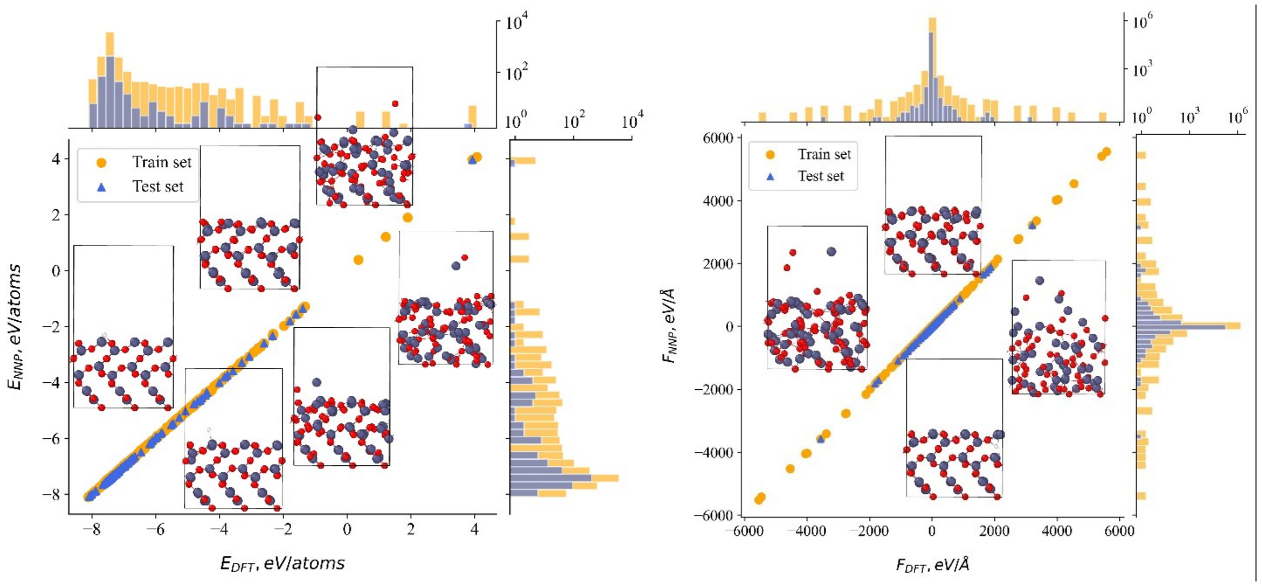

Figure S1. Correlation between DFT-calculated and NNP-predicted atomic energies (left) and forces (right) for the B_2O_3 systems, accompanied by histograms illustrating the distribution of their values. The structures shown below and the scatter points at the bottom provide representative examples corresponding to the distributions presented above.

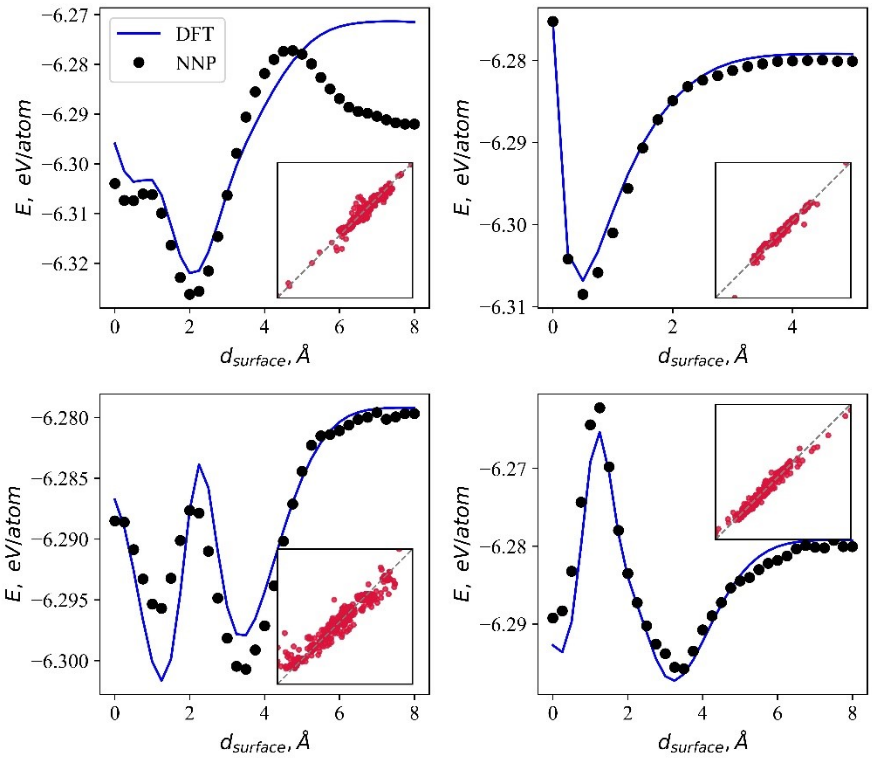

Figure S2. Energy profiles for the removal of a boron atom from the B(100) surface (top left) and for deuterium adsorption as a function of distance from different surface sites: top site (top right), Hollow 2 (bottom left), and Hollow 1 (bottom right). Insets in each subplot show the correlation between reference DFT forces and those predicted by the HDNNP model.

Table S3. RMSE values for atomic energies and forces predicted by the HDNNP model for the boron surfaces, compared to DFT reference data.

B-D system	$E_{RMSE,}$ eV/atom	F_{RMSE}, eV/A
B-site	8.96	145.37
D-Top	1.12	97.86
D-Hollow1	3.23	123.1
D-hollow2	4.19	117.19

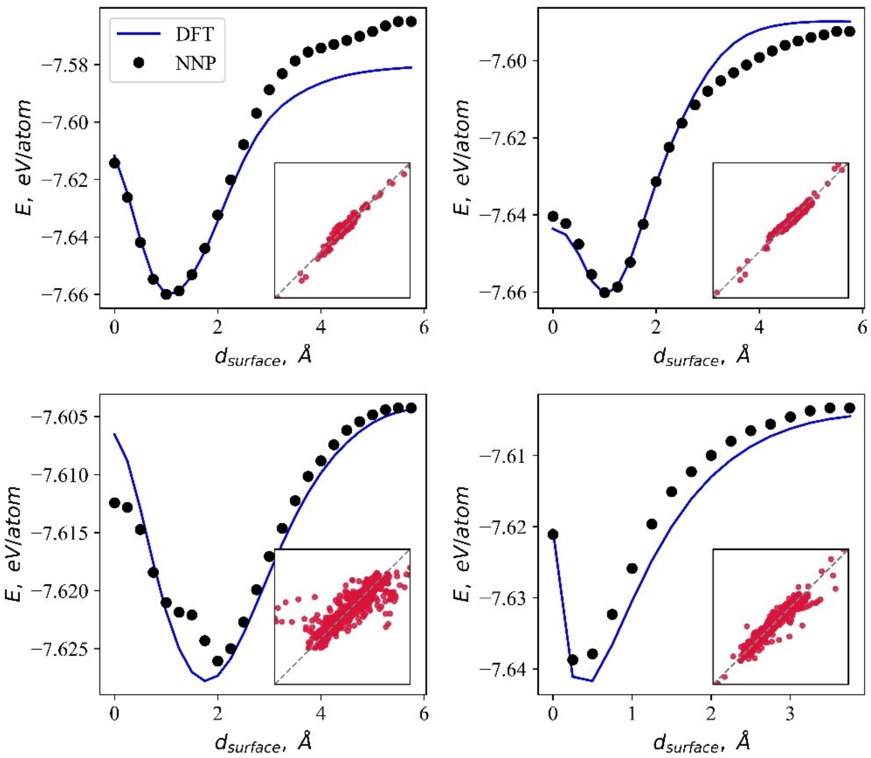

Figure S3. Energy profiles and force correlation graphs for the removal of a boron (top left) and oxygen (top right) atoms from the B_2O_3(001) and for deuterium adsorption as a function of distance from different surface sites: Hollow (bottom left), and top (bottom right). Insets in each subplot show the correlation between reference DFT forces and those predicted by the HDNNP model.

Table S4. RMSE values for atomic energies and forces predicted by the HDNNP model for the B_2O_3 surfaces, compared to DFT reference data.

B_2O_3 system	$E_{RMSE,}$ eV/atom	F_{RMSE}, eV/A
O-site	7.64	152.64
B-site	3.31	204.5
D-Hollow	1.69	154.17
D-Top	2.83	143.32

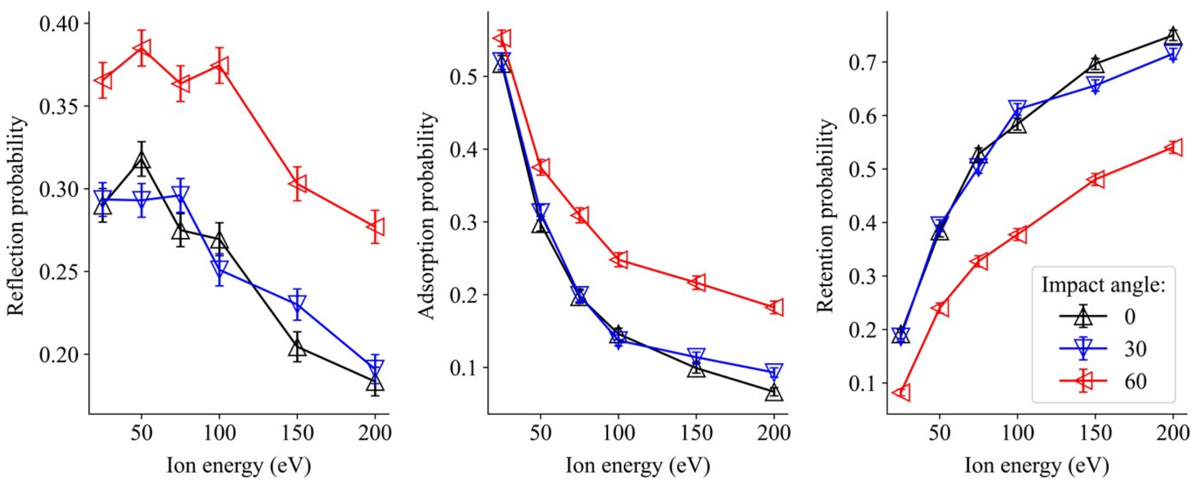

Figure S4: Deuterium atoms after a B_2O_3 surface collision: Probabilities of reflection (left), adsorption (middle), and retention (right).

References

1 M. Gastegger, L. Schwiedrzik, M. Bittermann, F. Berzsenyi and P. Marquetand, wACSF - Weighted atom-centered symmetry functions as descriptors in machine learning potentials, *J. Chem. Phys.*, 2018, **148**, 241709.

2 A. Singraber, J. Behler and C. Dellago, Library-Based LAMMPS Implementation of High-Dimensional Neural Network Potentials, *J. Chem. Theory Comput.*, 2019, **15**, 1827–1840.
